# The Twazon Arabic Weight Loss App: App-Based Intervention for Saudi Women With Obesity

**DOI:** 10.2196/10923

**Published:** 2019-05-28

**Authors:** Aroub Alnasser, Janet Kyle, Najla Aloumi, Abdulrahman Al-Khalifa, Debbi Marais

**Affiliations:** 1 Food Science and Nutrition Dept King Saud University Riyadh Saudi Arabia; 2 Institute of Applied Health Sciences University of Aberdeen Aberdeen United Kingdom; 3 Warwick Medical School University of Warwick Coventry United Kingdom

**Keywords:** obesity, weight loss, mobile applications, smartphone, obesity management, mHealth

## Abstract

**Background:**

By 2022, it is estimated that the rate of female obesity (78%) in Saudi Arabia will almost double that of males (41%). Despite being mainly attributed to poor diet, sedentary lifestyle, and a lack of health awareness, behavioral modification interventions are relatively new to the population; bariatric surgery continues to be the treatment of choice for comorbidities. However, neither pre nor postoperative diet and exercise are promoted. Evidence-informed mobile health (mHealth) weight loss apps and interventions may be an effective tool for delivering a culturally relevant intervention.

**Objective:**

This study aimed to determine the feasibility of a weight loss intervention that tests the effectiveness of Twazon, an originally designed Arabic weight-loss app that promotes lifestyle modification specific to Arab populations.

**Methods:**

A pre-post single‐arm pilot study was carried out among a sample of 240 overweight volunteer Saudi women residing in Riyadh, Saudi Arabia who used the Twazon app over a 4-month period. Anthropometric, diet, and physical activity measures were assessed 3 times: baseline, 2-months and 4-months; frequency of app use and system usability were evaluated during the 2 latter data collection periods. Repeated measures analysis of variance was used to identify changes over time.

**Results:**

A total of 40 participants completed the 4-month intervention with an attrition rate of 83%. An evaluation of the frequency of app use fostered 2 groups: engaged users (65%) and unengaged users (35%). At 4 months, the engaged users experienced more successful outcomes; body weight was lowered on average by 1.3 (SD 0.6) kg (*P*=.18), waist circumference (WC) was reduced by 4.9 (SD 1.1) cm (*P*<.001), and daily energy consumption was decreased by >600 calories (*P*=.002). Unengaged users experienced minor changes in body weight, WC, and reduced energy intake.

**Conclusions:**

The findings have demonstrated that engagement with the Twazon app renders positive changes in body weight, WC, and energy intake. mHealth weight loss apps and interventions have the potential to be effective in promoting weight loss and healthy lifestyle modification in Saudi Arabia and similar populations.

## Introduction

### Background

The prevalence of obesity in the Gulf region has become a serious public health issue, with the Kingdom of Saudi Arabia (KSA) having the highest prevalence rates at almost 35% [1]. In line with the global trend, Saudi women are affected by obesity more than men with current rates at 42% and 31%, respectively [2]. Projection studies on the increasing rate of obesity have anticipated a steady rise for both genders but more so for women; by 2022, 78% of females compared with 41% of males will be obese [3]. Studies have attributed the growing epidemic for Saudi women to a variety of factors, including low physical activity (PA), restrictive social norms based on gender inequality, and a transitioning diet [1]. The recent nutrition transition in Saudi Arabia is characterized by shifts in dietary patterns toward increased consumption of sugar, fat, and refined foods and can be linked to modernization, urbanization, economic development, and increased affluence within the population. Sedentary, inactive lifestyles are also more common under these conditions [4]. Despite this, research in the KSA has continued to focus on the descriptive analysis of age and gender differences rather than the effects of weight loss interventions [5].

Multicomponent lifestyle interventions aimed at modifying daily dietary and physical habits are effective in weight loss and weight management [6]. However, bariatric surgery is the preferred option in the KSA with 15,000 annual operations [7]; successful long-term outcomes are not enduring [8] and are associated with an absence of lifestyle and diet modification postsurgery [9]. A more sustainable and appropriate intervention design that includes behavior modification is needed for Saudis with obesity [6,10,11]. The worldwide popularity of mobile technology has provided a unique platform for delivering health information to the public via mobile apps, some of which have shown to positively promote weight loss and lifestyle modification [12]. In the KSA, the use of mobile phones that have an operating system capable of running downloaded apps (smartphones), has become ubiquitous [13]. There are many commercial Arabic weight loss apps available; however, an Arabic app screening [14] revealed that these apps show low adherence to evidence-informed practices (EIPs) for achieving weight loss and cultural insensitivity to diet and PA norms [14]. To fill the gap, the development of the Twazon Arabic weight loss app [15] was evidence informed and culturally adapted to meet the needs of its target audience.

### Objectives

As the first app-based weight loss intervention in the region, this pilot study aimed to evaluate the effectiveness and feasibility of a complex weight loss intervention that utilized the Twazon app as its main component. Developed over several phases [15,16], the primary objective of this app-based intervention was to facilitate weight loss through diet and PA modification. Over a 4-month period, outcomes of change in nutritional status, including weight loss, body mass index (BMI) and waist circumference (WC), as well as lifestyle habits including energy intake, Mediterranean diet (MD) adherence, and PA, were monitored.

## Methods

### Study Design

A pre-post single‐arm pilot study with 3 measurement points, this study was carried out to evaluate the effectiveness and feasibility of delivering a weight loss app intervention to overweight and obese Saudi women residing in Riyadh, Saudi Arabia.

### Participants and Recruitment

Recruitment was carried out online via social networks such as Twitter and by placing posters at various locations in Riyadh, the KSA. Interested participants completed a Web-based screening questionnaire regarding background information, medical history, and current physical status. Eligibility requirements included the following: gender (female), residency in Riyadh, age (≥18 years), BMI (≥25), stability of weight (<2 kg variation in 3 months), no current weight loss program or weight-affecting medication, smartphone ownership, interest in losing weight, and consent to participate. Participants were excluded if they were ever diagnosed with medical conditions such as diabetes and cancer or if already pregnant, trying to get pregnant, or lactating. Participants who met the requirements were invited via short message service (SMS) text messaging to participate.

### Measures

Anthropometric status, diet, and PA were assessed by trained researchers. Height and weight were measured to the nearest 0.1 cm and 0.01 kg, respectively, using the KERN electronic weighing scale (model MPB-P with stand). BMI was calculated as weight (kg)/height (m) using the World Health Organization (WHO) [17] cutoff points of 25.0 to 29.9 kg/m^2^ for overweight and ≥30 kg/m^2^ for obesity. WC was taken at the approximate midpoint between the lower margin of the last palpable rib and the top of the iliac crest [18] using a Seca 201 measuring tape to the nearest 0.1 cm; the WHO cutoff points define ≥80 cm as *increased risk of metabolic complications* and ≥88 cm as *substantially increased risk of metabolic complications* [18].

Total energy (kcal) average was calculated from two 24-hour dietary recalls on nonconsecutive days using the Automated Self-Administered 24-hour dietary recalls (ASA24) system [19]. To determine the nature of a participant’s diet, a culturally adapted version of the validated 14-item MD questionnaire was used. Alcohol consumption is forbidden in the KSA; therefore, the question pertaining to wine was eliminated, and each participant received 1 point to properly calculate the MD score (MDS). The MDS is calculated by adding up each point given, with a maximum of 14. The level of adherence was defined as follows: score 0 to 5, lowest adherence; score 6 to 9, average adherence; and score ≥ 10, highest adherence [20].

A culturally-relevant self-administered PA questionnaire [21] was used to assess an individual’s level of PA in terms of total metabolic equivalent task values (METs); METs levels are defined as *light* (<3 METs), *moderate* (3 to 6 METs), and *vigorous* (>6 METs) *intensity* [22]. A total of 60 min of moderate-intensity PA daily (eg, biking 10 to 12 miles per hour [mph] and walking 3 to 4 mph) can facilitate weight loss and maintenance for people with obesity or overweight [5]. The MET-min cutoff point of ≥1680 MET-min per week (60 min per day×7 days per week×4 METs) was used [23] to determine whether the recommendation was met.

The frequency of app use was heuristically determined through an algorithm (see below) created for Twazon that best calculated user activity or the frequency of user updates on certain measures (eg, diet, PA, and weight); time-stamped participant data were continuously downloaded to a secure server using the following: *Number of Active Days/ Two Weeks Count=Frequency of Use.*

The *number of active days* was calculated by adding up the number of days users uploaded their personal updated data. The *2 weeks count* was calculated by dividing the number of days since registered by 7 and then again by 2. These 2 figures were then computed as shown above to determine *frequency of use*. A score of 14 or less was indicative of how many times users updated their info in the app; a score under 1 meant less than 1 update every 2 weeks, a score of 1 meant at least 1 update every 2 weeks, and a maximum score of 14 meant at least 1 update every day. Frequency of app use was calculated every 2 weeks and then averaged at the end of the intervention.

To determine the overall usability of the tool, the 10-item System Usability Scale (SUS) questionnaire [24] was given to those participants who were labeled as engaged. Final SUS scores are linked with adjective ratings and acceptability ranges with a score of >70 being considered as *acceptable* [25,26].

### Intervention Implementation Procedure

A total of 3 assessments with a duration of 45 to 60 min were carried out at baseline, 2 months, and 4 months at 4 different locations in Riyadh; a voucher for 25 Saudi Arabian Riyals (or £5) or a small gift was given for participation. At the first assessment, individuals were interviewed and tutored on how to use the app. After completing the 2 ASA24 dietary recalls, participants were sent a link via SMS or WhatsApp to download the app, input personal information, and begin using the tool. Measures (refer to the subheading Measures) were evaluated at baseline, 2 months, and 4 months, with the exception of the SUS and frequency of app use scores, which were analyzed during the 2 latter assessments; height was measured at baseline. Reminders to attend the 2 month and 4 month assessments were also sent via SMS text messaging or WhatsApp.

### Statistical Analysis

Changes in the frequency of app use were evaluated using a repeated measures analysis of variance. Assessing outcome measures for within-group differences was carried out by the time-by-group interaction effect, whereas between-group differences were attained through contrastive analysis. The *P* value significance was set at *P*<.05 and a Bonferroni correction was used. All analyses were performed using the SPSS version 24 (SPSS Inc). Power calculations for sample size indicated that 165 participants would provide 90% power to detect a difference in means of 7.1 kg. This translates to a clinically meaningful change of 10% weight loss using a paired t-test with a 0.05 2-tailed significance level. Assuming a 30% attrition rate, a sample of 215 participants was estimated.

### Ethics

Ethical approval was obtained from the University of Aberdeen College of Life Sciences Ethics Review Board and the Scientific Research Ethics Committee in Riyadh, at King Saud University. This study complies with the WHO guidelines for the reporting of health interventions using mobile phones [27].

## Results

### Baseline Participants

**Figure 1:**
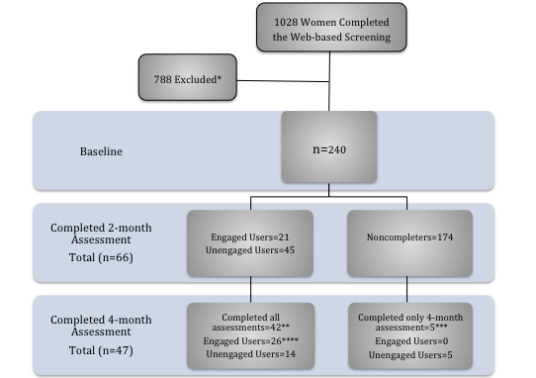
Flow of study participants. *Most common reason for exclusion was having a medical condition such as hypothyroidism. **Two participants were excluded because of being on a new diet. ***Five participants’ data were not included in the final analysis because of failure to attend the 2-month assessment. ****26= 21 users who stayed engaged +5 previously unengaged who became engaged at 4-months.

Out of 1028 interested Saudi women, 240 were eligible and were invited to participate in the intervention. Figure 1 shows the flow of participants at baseline, 2 months, and 4 months.

Baseline participants were clinically obese with an average weight of 81.8 (SD 15.1) kg, WC of 89.0 (SD 11.2) cm, and BMI of 32.9 (SD 5.6) kg/m^2^. In total, 49% (117/240) of all participants with a WC >88 cm were classified as *at high risk* of metabolic complications, 29% (69/240) were *at risk*, and 22% (52/240) had a healthy WC (Table 1).

**Figure 2:**
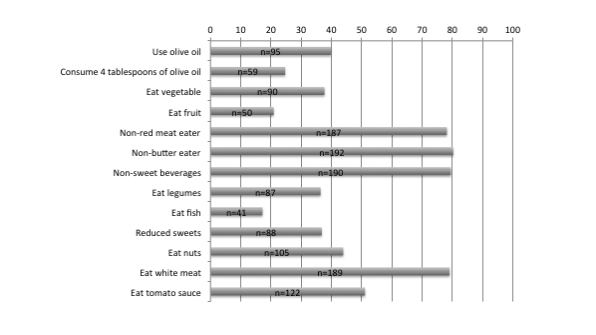
The number and percentage of the responses to Mediterranean diet questionnaire.

Energy intake was reported at 1692 (SD  751) calories per day, and the average MDS was 7.3 (SD 1.9), indicating medium MD adherence 68% (162/240). Over 75% of baseline participants reported little to no consumption of *red meat* (187/240), *butter* (192/240), or *carbonated/sugar-sweetened beverages* (SSBs; 190/240) and low consumption scores for fruit 21% (50/240) and fish 17% (41/240), (Figure 2). PA was deemed insufficient with almost 62% of baseline participants (146/236) not meeting the recommendation for weight loss; 38% (90/236) met the recommendation, and 4 women did not complete the PA questionnaire.

**Table 1:** Baseline characteristics of the 240 women.

Characteristics		Baseline values
Age (years), mean (SD)		31 (10)
Body weight (kg), mean (SD)		81.8 (15)
**Body mass index (kg/m2), mean (SD)**		**32.9 (5)**
	Overweight (25.0-29.9), n (%)	84 (35)
	Obese (≥30), n (%)	154 (65)
**Waist circumference (cm), mean (SD)**		**89.0 (11)**
	Normal <80, n (%)	52 (22)
	Risk ≥80, n (%)	69 (29)
	High risk ≥88, n (%)	117 (49)
Energy intake (kcal/d), mean (SD)		1692 (751)
Total Mediterranean diet score (point), mean (SD)		7.26 (2)
**Level of adherence, n (%)**		
	Low (≤5)	48 (20)
	Medium (6-9)	162 (68)
	High (≥10)	29 (12)
Physical activity (METs^a^-min/week), median (25^th^-75^th^ percentile)		1109 (334-2570)
Met the Recommendation. ≥1680 METs-min/ week, n (%)		90 (38)
Less than 1680 METs-min/ week, n (%)		146 (62)

^a^METs: metabolic equivalent task values.

### Frequency of App Use Scores

The results of app usage scores were heuristically determined to comprise 2 groups: *engaged* and *unengaged* users (See Measures in Methods). The more frequent the input, the higher the app use frequency score; ≥1 was labeled as engaged and <1 as unengaged. Users had to have updated their information in the app at least 8 times during the 16-week period to be considered engaged, which translates to a minimum of 1 update every 2 weeks. Of the 26 total engaged participants, 5 were labeled as unengaged at the 2-month assessment, but they increased their usage in the last 2 months, which relabeled them as engaged at the 4-month assessment. No participant went from being labeled as engaged (at 2 months) to unengaged at 4 months.

### Effects of 4-month Intervention

#### Anthropometric Changes

Engaged users lost 1.3 (SD 0.6) kg of body weight (*P*=.03), significantly reduced WC by 4.9 (SD 1.1) cm (*P*<.001), and lowered BMI by 0.6 (SD 0.2). Unengaged users lost 0.2 (SD 0.8) kg of body weight, reduced their WC by 3.0 (SD 0.8) cm, and exhibited no change in BMI (Table 2).

#### Diet and Lifestyle Changes

##### Energy Intake

Engaged users significantly reduced their energy intake by 672 calories per day (*P*=.002; see Multimedia Appendix 1), consuming 1470 (SD 535) calories per day, thereby meeting the reduction requirement [5] for decreasing weight-related risks. Unengaged users lowered their energy intake by 204 calories per day, consuming 1644 (SD 453) calories per day, but they failed to meet the requirement for weight loss (Table 2).

##### Mediterranean Diet

Engaged and unengaged participants had a mean MDS of 8.65 (SD 2.2) and 8.00 (SD 2.7), respectively, indicating a statistically significant improvement in overall MD adherence (*P*=.003). High MD adherence was achieved by 40% (16/40) of all participants at 4 months, compared with 12% (6/40) at baseline. Adherence to fruit (Q4; *P*=.005) and vegetable (Q3; *P*=.02) consumption was improved by all participants collectively. The engaged participants significantly lowered consumption of sweets and improved MD adherence (*P*<.001), whereas the unengaged participants increased their intake and lowered adherence. Table 3 shows mean MDS and adherence to MD questions. Multimedia Appendix 2 shows *P* values and adherence to MD questions (See Multimedia Appendix 2).

##### Physical Activity

At baseline, 69% (18/26) of engaged participants and 61% (8/14) of unengaged participants did not meet the PA recommendation, labeling them as inactive. The most common reason given for physical inactivity at any of the 3 assessments was *lack of time*. By 4 months, the engaged participants and unengaged participants increased their PA by 112 and 48 METs-mins per week, respectively (Table 2); however, no statistically significant changes were achieved by either of the 2 groups and neither met the recommendation (Table 4).

**Table 2:** Summary of effect results after the 4-month intervention.

Changes	Engaged		Unengaged		*P* values	
	Mean (SD)	%	Mean (SD)	%	Within‐group difference	Between‐group difference
Body weight (kg)	–1.3 (0.6)	–2	–0.2 (0.8)	0	.18	.34
Waist circumference (cm)	–4.9 (1.1)	–5	–3 (0.8)	–3	<.001	.35
Body mass index (kg/m^2^)	–0.6 (0.2)	–2	0 (0.2)	0	.18	.27
Energy (kcal/d)	–672 (283)	–31	–204 (403)	–11	.002	.30
Adherence to Mediterranean diet score	1.3 (0.5)	18	0.54 (0.5)	7	.003	.39
Physical activity	112 (669)	8	48 (148)	3	.77	.49

**Table 3:** Adherence to Mediterranean diet score.

Level of adherence	Engaged (n=26)		Unengaged (n=14)	
	Baseline, n (%)	4 months, n (%)	Baseline, n (%)	4 months, n (%)
Low (≤5)	4 (15)	3 (12)	2 (15)	3 (23)
Medium (6-9)	19 (73)	13 (50)	8 (62)	4 (31)
High (≥10)	3 (12)	10 (39)	3 (23)	6 (46)

**Table 4:** Success rate of adherence to recommended daily physical activity.

Physical activity	Engaged (n=26)		Unengaged (n=14)	
	Baseline, n (%)	4 months, n (%)	Baseline, n (%)	4 months, n (%)
Met the recommendation ≥1680 METs-min/ week	8 (31)	7 (27)	5 (39)	5 (39)
Less than 1680 METs-min/ week	18 (69)	19 (73)	8 (62)	8 (62)

### System Usability Scale

An average of the SUS scores calculated at 2 months and 4 months was determined for all participants. Engaged and unengaged users had an average SUS score of 69.3 (SD 10) and 64.3 (SD 8.6), respectively, indicating a marginally high acceptability rating (approximately >63) closer to an adjective rating of *good* (approximately >71.4) than of *ok* (approximately >50.9). A more in-depth analysis was carried out in a triangulated study on the relationship among actual user experiences, that is, the SUS score from this study, evidence-informed requirements [14], and potential user expectations [16], to explore the users’ primary preferences and suggested improvements of the Twazon weight loss app [28].

## Discussion

### Principal Findings

This pilot study aimed to evaluate the effectiveness of the Twazon Arabic weight loss app and to determine the feasibility of implementing it in a weight loss intervention among overweight and obese Saudi women. Due to a limited sample size and some methodological concerns, no concrete conclusions can be made about the effectiveness of the Twazon app. This study also reveals that the intervention may not be feasible because of low retention, despite successful recruitment and no significant differences found in baseline data between noncompleters and completers. A detailed discussion of these issues and how they might be avoided follows in this section.

### Effectiveness of the Twazon App

System usability was deemed to be of marginally high acceptability and those participants who engaged with the app more frequently, also experienced more successful outcomes. The engaged users lost an average of 0.2 (SD 0.1) kg by 2-months, and an additional 1.1 (SD 0.5) kg by the end of the intervention, with a total loss of 2% of baseline weight. This did not meet the 10% of clinically recommended weight loss for reducing risk factors associated with obesity, however, similar findings [29] in a smartphone-based weight-loss intervention showed an average loss of 1.8 kg after 6-months, indicating sustainable gradual weight loss over time. By 4-months, the engaged users significantly reduced WC by 4.9 (SD 1.1) cm (*P*<.001), going from a *substantially increased risk* to an *increased risk* for metabolic complications, and representing progress toward clinically significant weight-loss.

Energy intake and daily diet choices seem to have been positively influenced by increased interaction with the app. By the end of the intervention, engaged users reported an average intake of 1470 (SD 535) calories per day**,** representing the recommended daily intake of 1200-1600 calories per day for achieving weight loss [30]. Although overweight and obese women often underreport their energy intake [31], the ASA24 can be a useful tool for determining the dietary intake of this population; Widamen and colleagues [32] found only a 5% difference between energy reported and actual energy consumed. Despite the associations between obesity and the issue of underestimating, this study considered the process of self-monitoring to be more important than the accuracy of reported dietary intake [33].

MD adherence revealed that the rate of positive MD adjustment increased after 4-months for those who engaged with the app. Engaged participants reported consuming very little red meat, butter or carbonated and SSBs at baseline and 4-months, with small improvements made for the latter two. At 4-months the engaged participants were more successful at increasing or maintaining MD adherence with the most notable declines in adherence being for the consumption of sweets and SSBs. Changing trends in Saudi food choices that may contribute to the obesity problem should be considered for their role in health-related outcomes; for instance, some highly popular, foreign foods such as high-fat processed cheese products and spreads [34,35] may not have been reported because of their absence on the MD questionnaire. The low reporting of SSB consumption in this study is also questionable as research conducted in the KSA reported that at least 46% of adults are consuming one or two soft drinks, energy drinks and/or sweet beverages a day [36-38]. A preexisting knowledge of SSBs being associated with increased obesity [39] may have had an influence on low-reporting, however, it is also possible the question was not understood to include fruit drinks, juices or syrup-flavored coffee beverages which contain excessive amounts of sugar [40-42].

The unengaged users experienced some progress in diet by decreasing caloric intake by 204 (SD 403) calories per day and lost 0.2 (SD 0.8) kg of body weight. These results could be attributed to awareness-induced outcomes. A review study [43] indicated that participants were more conscious of certain diet and lifestyle habits after the intervention, despite not having used the app because of the information they received during the assessments and questionnaires. In terms of PA improvements, the lack of significant differences was not unexpected for 2 main reasons: first, a meta-analysis of weight-loss apps showed no significant changes in PA [12], and second, three-quarters of Saudi women are characterized as *insufficiently active* [44], ranking as the least physically active worldwide. Causes for the lack of PA in the KSA include an absence of locations designed for general PA such as sidewalks or green spaces and very limited options for female-oriented PA; only high-cost gyms or sports clubs exist which means that normal daily walking or aerobic movement is not a common activity for either gender [45].

### Feasibility of the Twazon Intervention

Although high recruitment rates were achieved (n=240 participants, or 10% of the original recruitment target) only 17% of the sample was retained. It is difficult to identify the exact reasons for attrition; however, there are several possibilities. Seed and colleagues suggest that high recruitment rates have the potential to have a negative effect on retention rates, such as individuals initially participating but not committing to the full duration of an intervention [46]. Whether or not a participant had the intent to commit or was actually prepared to devote the necessary work to begin losing weight was not assessed before beginning the current study. Being interested in losing weight was a requisite but may not have been an adequate indicator of what a participant was willing to invest in terms of behavior change and self-monitoring. A readiness questionnaire that also indicates what the commitment potentially could entail in terms of time and effort might assist in evaluating this. Participants who are new to lifestyle modification or weight loss may have felt unprepared, thereby affecting retention [47].

High attrition may be also explained by motivation; very little is known about what drives a Saudi man or woman to participate in a weight loss intervention. In line with this, an aversion to goals perceived as not being robust enough or disappointing [48,49] may have demotivated participants. During recruitment, many women reported losing interest in the intervention due to dissatisfaction with the goal of a 10% loss of total body weight [50,51]. Other app-based weight-loss interventions have found that many participants with obesity discontinued app use if the initial amount of weight lost was unsatisfactory [52,53]. Although Foster [48] and other studies [49,54] found that a weight-loss goal of 22-34% was expected and perceived as more motivating, attainment of 50% of said goal resulted in positive physical and psychosocial effects. If less-than-satisfactory weight loss goals can still be beneficial to obese individuals, then there may be a need for cognitive intervention aimed at improving personal body image and reducing the importance of weight as a number.

The amount of personal contact had with researchers could also have had an effect on a participant’s adherence to the Twazon intervention. Weight loss interventions delivered via mobile phone that also included daily personal contact by those carrying out the intervention were found to be more effective than a stand-alone app [55]. Although there have not been any studies conducted in the KSA assessing the effect of personal contact, Aroian and colleagues’ longitudinal study [56] in the United States states that explored the efficacy and inefficacy of strategies used to recruit and retain Arab Muslim immigrant women and their children for research. Their results indicated that personal contact with data collectors that they trusted and had good rapport with, was the most important motivator for adherence to the study. This should be explored by future research, as these findings may hold true in Arab nations like the KSA, and perhaps in other high-context communication cultures [57,58] that place substantial value on the community.

### Strengths and Limitations

The Twazon weight loss app intervention is, to the best of our knowledge, the first of its kind in the Gulf region, and unique in its focus on adult Saudi women, in its use of an original Arabic weight loss app, and in its development being in compliance with EIPs and behavior change techniques for weight-loss. This study is not without its limitations, which include: (1) a lack of control group, (2) recruitment that combined traditional methods with social media, (3) discrepancies between changes in weight and WC, (4) normal fluctuations in body weight changes was not considered, (5) possible underreporting of energy intake, and finally, (6) no validated index of app use was available. Future research should be aware of these limitations when designing protocol for similar studies so that more conclusive evidence can support the findings.

### Conclusions

The study set out to evaluate the effectiveness of the culturally-adapted and evidence-informed Arabic Twazon app which was developed to promote weight loss through lifestyle modification, and to determine the feasibility of using said app in an intervention conducted among obese and overweight Saudi women. Although some positive outcomes of change were observed, limited sample size did not allow for any concrete conclusions to be established from the current study’s findings, and therefore, the effectiveness of Twazon cannot be confirmed. Whether the complexity of the Twazon app’s multicomponent features was encouraging of weight loss or overly demanding on the participants is also still unknown. In regards to the feasibility of the Twazon app intervention, retention was challenging, and for this reason it is not certain if app-enabled mobile phone technology should be used in weight-loss interventions among the target population; researchers should conduct more in-depth exploratory analysis of potential issues before designing clinical trials. Regarding local policy in the KSA, a substantial increase in the availability of PA-promoting environments and the integration of health-and-diet education needs to be implemented for all citizens of all ages if the obesity epidemic is to be effectively managed. Future studies are sorely needed in and around the Middle East as well as among similar populations found outside the region.

## Appendix

Multimedia Appendix 1 Repeated measure analysis of variance (ANOVA) analyses of 4-month weight loss intervention for the engaged users (n=26) andunengaged users (n=14).

Multimedia Appendix 2 Results of repeated measure analysis of variance (ANOVA) of the Mediterranean diet Scores for the engaged users (n=26) and unengaged users (n=14).

## Abbreviations

ASA24: Automated Self-Administered 24-hour dietary recalls
BMI: body mass index
EIP: evidence-informed practice
KSA: Kingdom of Saudi Arabia
MD: Mediterranean diet
MDS: Mediterranean diet score
METs: metabolic equivalent task values
mHealth: mobile health
mph: miles per hour
PA: physical activity
SMS: short message service
SSB: sugar-sweetened beverage
SUS: System Usability Scale
WC: waist circumference
WHO: World Health Organization
