# Supplementary material for: The Twazon Arabic Weight Loss App: App-Based Intervention for Saudi Women With Obesity
Source: JMIR Mhealth Uhealth. 2019 May 28;7(5):e10923. doi: 10.2196/10923 (PMC6658271; doi:10.2196/10923)
Supplement: Multimedia Appendix 1 [file mhealth_v7i5e10923_app1.pdf]

Multimedia Appendix 1

Repeated measure ANOVA analyses of 4-month weight loss intervention for the engaged users (n=26) and unengaged users (n=14)

	Baseline Values		2-Months Values		4-Months Values		Time[a]	Time * group[b]
**REPEATED MEASURES RESULTS**	**Unengaged**	**Engaged**	**Unengaged**	**Engaged**	**Unengaged**	**Engaged**	**p-value**	**p-value**
**Body Weight, Kg**	80.6 ± 12.5	83.4 ± 15.2	80.5 ± 12.5	83.2 ± 15.3	80.4 ± 11.7	82.1 ± 15.8	0.183	0.338
**WC (cm)**	89.1 ± 10.4	91.1 ± 7.7	87.2 ± 10	88.9 ± 8.6	86.1 ± 9.6	86.2 ± 8.8	<0.001	0.351
**BMI (kg/m2)**	32.3 ± 3.7	33.5 ± 4.6	32.3 ± 3.8	33.4 ± 4.7	32.3 ± 3.5	32.9 ± 4.8	0.182	0.272
**Energy intake (kcal/d)**	1848 ± 856	2142 ± 818	1563 ± 487	1486 ± 549	1644 ± 453	1470 ± 535	0.002	0.295
**TOTAL MDS**	7.46 ± 2.2	7.35 ± 1. 7	8.62 ± 1.6	8.15 ± 2.1	8.0 ± 2.7	8.65 ± 2.2	0.003	0.388
**Physical activity (METs-min/ week)**	1749 ± 1765	1390 ± 1296	2379 ± 2794	1268 ± 1086	1797 ± 1617	1502 ± 1965	0.772	0.498

[a] P-value tests for a significant within-group difference at all time points

[b] P-value tests for a significant between-group difference at all time points
