# Supplementary material for: The Twazon Arabic Weight Loss App: App-Based Intervention for Saudi Women With Obesity
Source: JMIR Mhealth Uhealth. 2019 May 28;7(5):e10923. doi: 10.2196/10923 (PMC6658271; doi:10.2196/10923)
Supplement: Multimedia Appendix 2 [file mhealth_v7i5e10923_app2.pdf]

Multimedia Appendix 2

Results of repeated measure ANOVA analysis of the MD Scores for the engaged users (n=26) and unengaged users (n=14)

	Baseline		2 months		4 months		Time[a]	Time * group[b]
**Variable**	**Non engaged**	**Engaged**	**Non engaged**	**Engaged**	**Non engaged**	**Engaged**	**p value**	**p value**
**Q1: Use olive oil**	**.46 ± .52**	**.50 ± .51**	**.62 ± .50**	**.42 ± .50**	**.46 ± .52**	**0.62 ± .50**	**0.868**	**0.15**
**Q2: How much olive oil**	**.38 ± .51**	**.23 ± .43**	**.31 ± .48**	**.12 ± .33**	**.38 ± .51**	**.23 ± .43**	**0.535**	**0.975**
**Q3: Yes vegetable**	**.31 ± .48**	**.46 ± .51**	**.69 ± .48**	**.65 ± .49**	**.38 ± .51**	**.65 ± .49**	**0.019**	**0.188**
**Q4: Yes fruit**	**.23 ± .44**	**.15 ± .37**	**.54 ± .52**	**.46 ± .51**	**.38 ± .50**	**.38 ± .50**	**0.005**	**0.92**
**Q5: No red meat**	**.77 ± .44**	**.85 ± .37**	**.77 ± .44**	**.88 ± .33**	**.85 ± .38**	**.85 ± .37**	**0.919**	**0.806**
**Q6: No butter**	**.69 ± .48**	**.81 ± .40**	**.77 ± .44**	**.92 ± .27**	**.77 ± .44**	**.92 ± .27**	**0.418**	**0.965**
**Q7: No sweet beverages**	**.85 ± .38**	**.88 ± .33**	**.92 ± .28**	**.92 ± .27**	**.69 ± .48**	**.92 ± .27**	**0.279**	**0.248**
**Q8: Yes legumes**	**.31 ± .48**	**.27 ± .45**	**.46 ± .52**	**.42 ± .50**	**.54 ± .52**	**.42 ± .50**	**0.087**	**0.912**
**Q9: Yes fish**	**.15 ± .38**	**.04 ± .2**	**.23 ± .44**	**.08 ± .27**	**.31 ± .48**	**.19 ± .40**	**0.146**	**0.905**
**Q10: Reduced sweets**	**.69 ± .48**	**.19 ± .40**	**.31 ± .48**	**.38 ± .50**	**.31 ± .48**	**.58 ± .50**	**0.616**	**0.001**
**Q11: Yes nuts**	**.38 ± .51**	**.50 ± .51**	**.54 ± .52**	**.58 ± .50**	**.62 ± .51**	**.65 ± .49**	**0.2**	**0.919**
**Q12: Yes white meat**	**0.69 ± .48**	**0.81 ± .4**	**.92 ± .28**	**.88 ± .33**	**.85 ± .38**	**0.85 ± .37**	**0.15**	**0.567**
**Q13: Yes tomato sauce**	**0.54 ± .52**	**.65 ± .49**	**.54 ± .52**	**.42 ± .50**	**.46 ± .52**	**.38 ± .50**	**0.101**	**0.344**
**TOTAL MDS**	**7.46 ± 2.2**	**7.35 ± 1.7**	**8.62 ± 1.6**	**8.15 ± 2.1**	**8.0 ± 2.7**	**8.65 ± 2.2**	**0.003**	**0.388**

[a] **P-value tests for a significant within-group difference at all time points**
[b] **P-value tests for a significant between-group difference at all time points**
